# Hospital physicians’ work stressors in different medical specialities: a statistical group comparison

**DOI:** 10.1186/s12995-015-0052-y

**Published:** 2015-02-25

**Authors:** Grit Tanner, Eva Bamberg, Agnessa Kozak, Maren Kersten, Albert Nienhaus

**Affiliations:** University of Hamburg, Work and Organisational Psychology, Von-Melle-Park 11, 20146 Hamburg, Germany; University Medical Center Hamburg-Eppendorf, Institute for Health Services Research in Dermatology and Nursing (IVDP), Martinistraße 52, 20246 Hamburg, Germany; Institute for Statutory Accident Insurance and Prevention in the Health and Welfare Services (BGW), Principles of Prevention and Rehabilitation Department (GPR), Pappelallee 33/35/37, 22089 Hamburg, Germany

**Keywords:** Work stressors, Physicians’ everyday work, Comparison of medical specialities, Emotional exhaustion, Irritation

## Abstract

**Background:**

Some studies on the occupational health of hospital physicians have found that working conditions have different effects on physician’s well-being and health in different medical specialities. There has been no comparative study of the effects of various work stressors in different specialities. This study aims to close this gap.

**Methods:**

German hospital physicians were asked about their working conditions and aspects of health. The short version of the Instrument for Stress-Related Job Analysis for Hospital Physicians was used to measure working conditions. Irritation and emotional exhaustion were used to assess health. Physicians were also asked for socio-demographic aspects, including their medical speciality.

**Results:**

Data from 763 hospital physicians were included in the analyses. Significant differences between medical specialities were demonstrated for time pressure, uncertainty, frustration about how work needs to be done and social stressors with patients. Physicians in internal medicine showed consistently high levels of stressors. Time pressure, frustration about how work needs to be done, and emotional dissonance were found to be significantly related to both aspects of health.

**Conclusions:**

The results showed that some medical specialities are more affected by specific stressors. It is therefore discussed how improvements can be implemented. Furthermore, it is illustrated which stressors are especially relevant for health. These relationships to health should be investigated in further research and in longitudinal designs to allow hints of causal relationships.

## Background

There have been several scientific studies on the working conditions of hospital physicians [1-7]. The considered aspects include job satisfaction [1], work-life balance [2], multitasking and work interruptions [4,5]. Thereby, relationships to health have often been examined.

How specific working conditions can affect employees’ health is described in the extension of the transactional stress model in work and organisational psychology [8]. Stress is seen as a process that arises by assessing and then mastering the situation (either problem- or emotion-focused). The starting points for this process are situation-related stressors (e.g., time pressure), person-related risk factors (e.g., illness), as well as situation-related resources (e.g., social support) and person-related resources (e.g., self-efficacy). Stressors are characteristics that increase the probability of stress-outcomes. Resources are characteristics that can be used to master demands [8]. Situation-related stressors and resources represent the work environment. Both influence the assessment and mastery of situations and – in unfavourable proportions – lead to stress-outcomes. Aside from the work environment, person-related factors influence this process and cause or prevent the stress-outcomes as well. On the other hand, stress-outcomes interact directly with the above mentioned starting points.

In accordance with the transactional stress model of work and organisational psychology, studies have investigated specific situation-related stressors and resources within hospital physicians’ work, as well as relationships to other health aspects. Longitudinal studies have found long-term effects on health aspects – particularly for resources. Especially, autonomy was shown to be related to depressive symptoms in long-term [9,10]. Several cross-sectional studies have investigated whether situation-related stressors, health aspects and their relationships differ with respect to other factors such as medical speciality. For example, one study has examined differences between emergency physicians and other physicians [11]. The results show that, for emergency physicians, poor collaboration with colleagues is more closely related to intention to quit and burnout than for other physicians. On the other hand, the relationship between worry about making errors and burnout was about the same in the two samples [11]. For work engagement and burnout in assistant physicians, one study showed that there were differences between the medical specialities [12]. A further study has also shown that there are differences between the different medical specialities with respect to the working time of hospital physicians in Germany [13].

These results illustrate that stressors may have different effects in different medical specialities. But there has not been any comparison of different specialities across various stressors that cover almost the whole physicians’ working day. The current study aimed to bridge this gap by examining various situation-related stressors during the daily work of hospital physicians. These are compared for different specialities, so that any improvements could be developed specifically for the specialities. Moreover, relationships between stressors and stress-outcomes are examined. The current study primarily focuses only on stressors, as including other factors would increase the complexity to an excessive degree.

## Methods

This project was entitled *Stress*-*Related Job Analysis for Hospital Physicians* (StArK) and was carried out by the Institute for Statutory Accident Insurance and Prevention in the Health and Welfare Services, University of Hamburg and University Medical Center Hamburg-Eppendorf, in order to examine hospital physicians’ working conditions and their relationships to health.

### Sample

Within the StArK project, hospital physicians throughout Germany were requested to evaluate their working conditions via questionnaire from February 2012 to December 2012. In addition, a link to the online survey was published in the newsletter of the official journal for German physicians (*Deutsches Ärzteblatt*). Thus, hospital physicians could take part in the survey independently of their hospitals. To ensure that the results were reliable, only medical specialities with more than ten participants were included.

### Measures

The short version of the “Instrument for Stress-Related Job Analysis for Hospital Physicians” (ISAK-K) [14] was used to measure specific stressors of hospital physicians’ work. This instrument includes seven different stressors. On the one hand, task-related stressors, such as time pressure and uncertainty (e.g., from insufficient information) as well as frustration about how work needs to be done were investigated. On the other hand, interaction-related stressors were considered, including problems in workflow due to physicians (supervisors and colleagues) and other professional groups (e.g., keeping to agreements, transmission of information), social stressors with patients and their families, and emotional dissonance (control of personal emotions). The stressors contain two to three items, which were rated on a 5-point Likert-scale.

Two aspects were selected to assess psychological health: Irritation [15] and emotional exhaustion [16]. Irritation describes work-related rumination after work. This includes additional effects on the state of mind (feeling of tension and irritability) [15]. Irritation represents a precursor to more serious psychological impairments. The eight items were answered on a 7-point Likert-scale, from 1 (*does not apply at all*) to 7 (*applies nearly completely*). Emotional exhaustion results in long-term from excessive efforts, both emotional and physical [17]. It is characterised by avolition and a feeling of weakness. One cause of emotional exhaustion may be that professional effort is not adequately rewarded (e.g., by appreciation) [18]. Emotional exhaustion was measured with six items [16], which were answered on a 7-point Likert-scale, from 1 (*never*) to 7 (*daily*). Furthermore, on the basis of other studies control variables were selected. These studies demonstrated effects related to the medical speciality [12,13], the professional position or age [10,19] and gender [10].

### Statistical analyses

The comparison of medical specialities was conducted with one-way analyses of variance (ANOVA). The Bonferroni procedure (post-hoc test) was used to identify the groups with significant differences. If variance heterogeneity was confirmed, the Welch test was applied to examine the differences, followed by the Dunnett-T3 procedure for the post-hoc tests. The Bonferroni correction was used to counteract the accumulation of α-error [20]. Thus the significant ANOVA results presented here correspond to the corrected p-value.

Multiple hierarchical regression analyses were used to investigate the relationships between all stressors examined and each health aspect. In the first step, control variables were included, to ascertain whether, independently of these, there still is a relationship between the stressors and the health aspect. In the second step, all task-related stressors were included, followed by interaction-related stressors in the third step.

## Results

In sum, data were collected from 817 German hospital physicians. The response rate of the acquisition via hospitals was 30.6% (705 hospital physicians). Additionally, with a response rate of 40.4% and a completion rate of 20.3%, 112 hospital physicians participated in the open online-survey. After excluding groups smaller than ten participants (e.g., ENT physicians, occupational physicians, ophthalmologists, and pathologists), 763 physicians were included in the analyses – split into eleven different medical specialities. Socio-demographic characteristics of the samples are presented in Table 1. Both samples are comparable with German physician statistics [21,22].

**Table 1:** **Sample characteristics**

	**Total sample (N = 817)**	**Study sample (n = 763)**
**Mean age**	**41.0 (SD 9.8)**	**40.7 (SD 9.8)**
Gender		
Female	44.2%	43.0%
Male	55.8%	57.0%
Main medical specialities		
Internal medicine	21.8%	23.3%
Surgery	18.2%	19.5%
Anaesthesia	15.8%	16.9%
Professional position		
Assistant physician	39.7%	40.8%
Consulting physician	18.9%	18.3%
Senior physician	32.7%	32.5%
Chief physician	8.7%	8.4%

### Comparison of specialities

**Figure 1:**
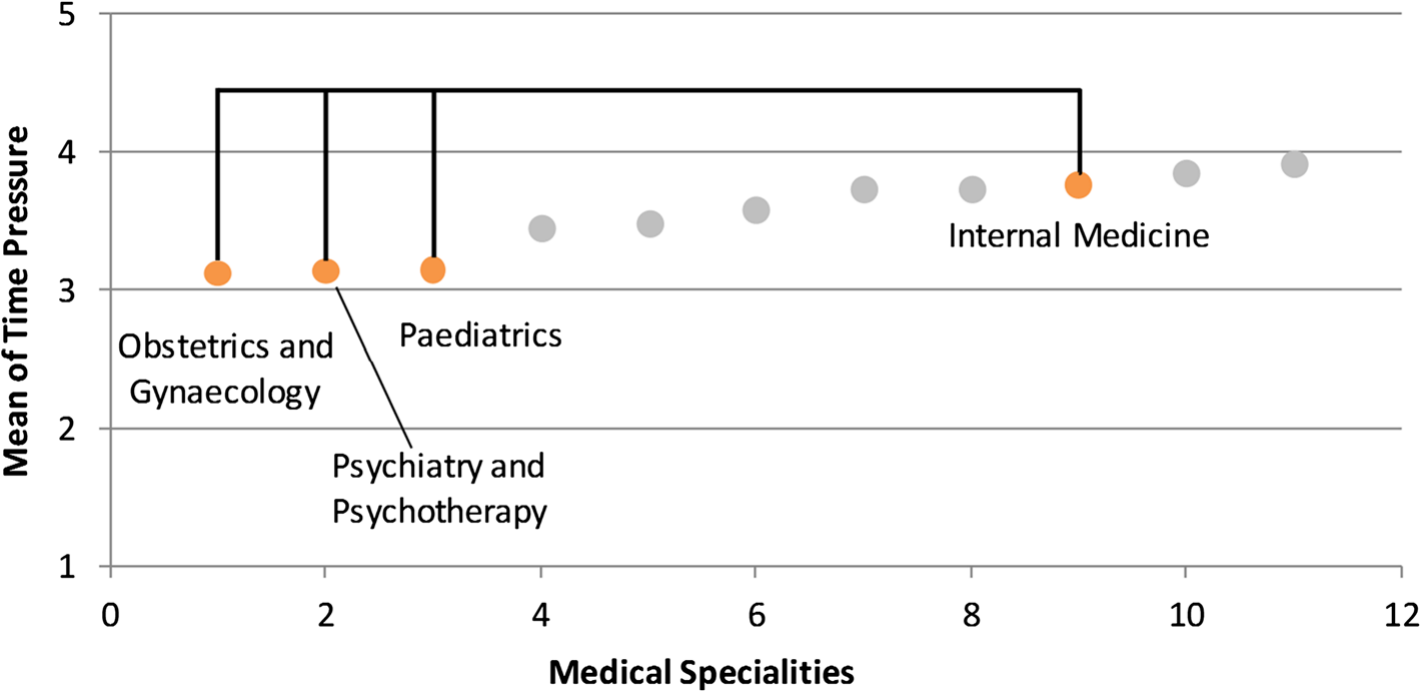
**Differences between internal medicine and other specialities in time pressure.** This is only a schematic figure of the differences found in time pressure. For detailed results use Table 2.

**Figure 2:**
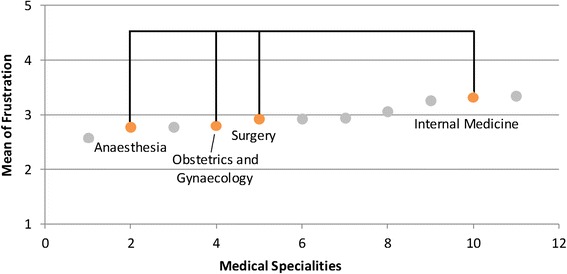
**Differences between internal medicine and other specialities in frustration.** This is only a schematic figure of the differences found in frustration about how work needs to be done. For detailed results use Table 2.

**Figure 3:**
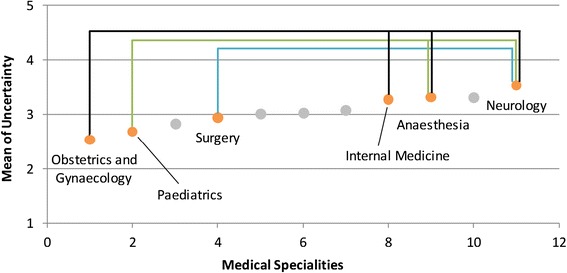
**Differences between gynaecology, paediatrics, or surgery and other specialities in uncertainty.** This is only a schematic figure of the differences found in uncertainty. For detailed results use Table 2.

**Figure 4:**
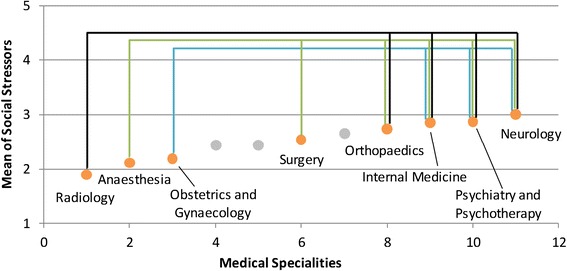
**Differences between radiology, anaesthesia, or gynaecology and other specialities in social stressors with patients/families.** This is only a schematic figure of the differences found in social stressors with patients/families. For detailed results use Table 2.

Various differences were found between the medical specialities with respect to the examined stressors (see Table 2; for a schematic overview see Figures 1, 2, 3, and 4). *Time pressure* and the *frustration about how work needs to be done* were rated higher by physicians in internal medicine than by those in obstetrics and gynaecology or in paediatrics (see Figures 1 and 2). Surgeons reported lower levels of *frustration* than physicians in internal medicine. Psychiatrists and psychotherapists reported lower levels of *time pressure* than physicians in internal medicine. *Uncertainty* was rated lower by physicians in obstetrics and gynaecology than by physicians in internal medicine, anaesthesia and neurology (see Figure 3). Moreover, physicians in paediatrics rated *uncertainty* lower than physicians in anaesthesia and neurology. Neurologists rated *uncertainty* higher than did surgeons. Several significant differences were found with respect to *social stressors with patients and their families* (see Figure 4)*.* Physicians in radiology, anaesthesia, and in obstetrics and gynaecology rated *social stressors* significantly lower than did physicians in internal medicine, neurology, and in psychiatry and psychotherapy. Physicians in orthopaedics and rehabilitation rated *social stressors* higher than did physicians in radiology and anaesthesia. Surgeons gave also higher rates for *social stressors* than did physicians in anaesthesia. On the other hand, for the stressors *problems in workflow due to physicians and other professional groups* and *emotional dissonance,* there were no significant differences between the ratings in the medical specialities.

**Table 2:** **One-way analyses of variance for the stressors by medical speciality**

	**Anaesthesia**	**Surgery**	**Obstetrics and gynaecology**	**Internal medicine**	**Paediatrics**	**Neurosurgery**	**Neurology**	**Orthopaedics**	**Psychiatry and psychotherapy**	**Radiology**	**Urology**	**F**
	**(n = 129)**	**(n = 149)**	**(n = 60)**	**(n = 178)**	**(n = 52)**	**(n = 20)**	**(n = 44)**	**(n = 46)**	**(n = 50)**	**(n = 23)**	**(n = 12)**	
Time pressure	3.59	3.49	3.13^a^	3.76^a,b,c^	3.15^b^	3.85	3.74	3.45	3.14^c^	3.74	3.92	3.93***
Uncertainty^1^	3.32^d,e^	2.94^f^	2.53^d,g,h^	3.27^g^	2.68^e,i^	3.33	3.53^f,h,i^	3.00	2.83	3.09	3.04	4.71***
Frustration about how work needs to be done	2.78^j^	2.93^k^	2.80^l^	3.33^j,k,l^	2.78	2.93	3.35	3.05	2.96	2.58	3.28	3.85***
Problems in workflow due to supervisors and colleagues	2.45	2.29	2.43	2.47	2.09	2.38	2.19	2.47	2.28	2.52	2.58	1.47
Problems in workflow due to other professional groups	2.45	2.45	2.65	2.71	2.30	2.55	2.65	2.67	2.44	2.72	2.63	1.84
Social stressors with patients/families	2.11^m, n, o, p, q^	2.54^m^	2.19^r,s,t^	2.85^n,r,u^	2.45	2.45	3.01^o,s,v^	2.75^p,w^	2.88^q,t,x^	1.89^u,v,w,x^	2.67	9.33***
Emotional dissonance^1^	2.52	2.41	2.33	2.54	2.15	2.43	2.48	2.75	2.97	2.28	2.71	1.96

N = 763. Post-hoc test: Bonferroni. ^1^Because of the heterogeneous variance, tested with the Welch-test and Post-hoc test: Dunnett-T3.

Significant group differences are marked with the same high-ranking letters: ^a^Internal medicine - Gynaecology ** ; ^b^Internal medicine - Paediatrics * ; ^c^Internal medicine - Psychiatry/Psychotherapy * ; ^d^Anaesthesia - Gynaecology *** ; ^e^Anaesthesia - Paediatrics * ; ^f^Surgery - Neurology* ; ^g^Gynaecology - Internal medicine*** ; ^h^Gynaecology - Neurology *** ; ^i^Paediatrics - Neurology ** ; ^j^Internal medicine - Anaesthesia *** ; ^k^Internal medicine - Surgery * ; ^l^Internal medicine - Gynaecology * ; ^m^Anaesthesia - Surgery** ; ^n^Anaesthesia - Internal medicine*** ; ^o^Anaesthesia - Neurology *** ; ^p^Anaesthesia - Orthopaedics ** ; ^q^Anaesthesia - Psychiatry/Psychotherapy *** ; ^r^Gynaecology - Internal medicine *** ; ^s^Gynaecology - Neurology *** ; ^t^Gynaecology - Psychiatry/Psychotherapy ** ; ^u^Radiology - Internal medicine *** ; ^v^Radiology - Neurology *** ; ^w^Radiology - Orthopaedics * ; ^x^Radiology - Psychiatry/Psychotherapy ***.

*p < .05; **p < .01; ***p < .001.

### Relationships between Stressors and Health

As can be seen in Table 3, all stressors – as well as the control variables age, gender, and professional position – showed significant correlations with the health aspects irritation and emotional exhaustion. There was no significant correlation between the medical speciality and the two health aspects. In the multiple regression analyses with control for age, gender, professional position and medical speciality, results were different. Of the task-related stressors, *time pressure* and *frustration about how work needs to be done* showed significant relationships to both irritation and emotional exhaustion. Of the interaction-related stressors, only *emotional dissonance* showed a significant relationship to both stress-outcomes. In addition, *problems in workflow due to other professional groups* were significantly related to emotional exhaustion. Moreover, beyond task-related stressors, interaction-related stressors explained 5 to 7% of additional variance in the stress-outcomes.

**Table 3:** **Hierarchical multiple regression analyses explaining irritation and emotional exhaustion**

	**DV: irritation**			**DV: emotional exhaustion**		
	***r***	**β**	**Δ** ***R*** ^**2**^	***r***	**β**	**Δ** ***R*** ^**2**^
*Step 1: Control variables*			.02**			.05***
Medical speciality	-.03	-.07*		.05	.00	
Professional position	-.12**	-.02		-.02***	-.15***	
Age	-.13***	-.04		-.18***	.01	
Gender	.11**	.12***		.10**	.07*	
*Step 2: Task-related stressors*			.21***			.32***
Time pressure	.37***	.16***		.48***	.28***	
Uncertainty	.36***	.05		41***	.02	
Frustration about how work needs to be done	.40***	.12***		.52***	.19***	
*Step 3: Interaction-related stressors*			.07***			.05***
Problems in workflow due to supervisors and colleagues	.27***	.08		.28***	.02	
Problems in workflow due to other professional groups	.28***	.03		.35***	.11**	
Social stressors with patients/families	.31***	.04		.36***	.03	
Emotional dissonance	.44***	.26***		.45***	.19***	

DV = dependent variable; *r* = Pearson correlation coefficient; β = standardised regression coefficient; Δ *R* ^2^ = changes in coefficient of determination *R* ^2^ to estimate source of variance. Numerical coding for control variables: medical speciality: 1 = Anaesthesia (sorted alphabetically) to 11 = Urology; Professional position: 1 = assistant physician (sorted in rising order), up to 4 = chief physician; gender: 1 = male, 2 = female.

*p < .05; **p < .01; ***p < .001.

## Discussion

Especially physicians in internal medicine rated stressors more highly than do physicians in other specialities. Assuming that specific stressors (e.g., stressors with patients, lack of information) are negatively related to work engagement [23,24], these results support the findings of a study with Dutch assistant physicians [12]. In this study, physicians in internal medicine showed less work engagement than colleagues in other specialities, combined with high emotional exhaustion [12]. However, the latter finding was not confirmed in the current study, as no relationship between medical speciality and emotional exhaustion was found. The differences with respect to the social stressors with patients and their families were predictable, if the differences in patient contact were considered for the different specialities. Specialities such as neurology, psychiatry and psychotherapy, and internal medicine inherently involve more communication with patients (e.g., for diagnosis) than in radiology or anaesthesia. Regarding uncertainty (e.g., consequences that are difficult to predict) and social stressors with patients, neurologists not only showed significant differences, but gave the highest ratings compared to the other specialities. As these stressors are presumably related to the medical problems in this speciality, it would be useful to investigate the role of resources (both, situation- and person-related) on stress-outcomes.

The results of the multiple regression analyses show that time pressure, frustration about how work needs to be done and emotional dissonance are related to different stress-outcomes. A prior study has already shown that time pressure is associated with a high feeling of stress in physicians [1]. The current results support this feeling perceived by physicians. The frustration about how work needs to be done means restrictions due to documentation and administration work. At least for surgeons and internal physicians, these tasks take up about one third of daily working hours [5]. This illustrates once again how important the relationship between frustration and stress-outcomes is. Thus, it seems to be useful to reduce high levels of frustration.

### Strengths and limitations

As the sample was relatively large, it was possible to compare eleven different specialities. However, a selection bias cannot be excluded. For example, it is possible that unusually stressed physicians took part in this survey. Despite that, the current sample corresponds to the physician statistics of the German Medical Association [21] and the German Federal Bureau of Statistics [22], with respect to age, gender and medical specialities. Another advantage of this study is that it considers two potential stress-outcomes. Nevertheless, the relationships between stressors and health aspects should be interpreted with caution. Because of the cross-sectional design, no causal interpretations are possible. Hints for causal conclusions can only be drawn from longitudinal studies or studies with experimental designs [25]. Moreover, it should be emphasised that several situation-related stressors were considered in the current study, which represent a large part of the hospital physician’s everyday work. Although the current analyses focused only on stressors, a further emphasis on resources should be considered because work-related resources are found to be relevant to health as well [26], as mentioned in the transactional stress model of work and organizational psychology.

### Practical implications

Various approaches will be discussed to reduce the stressors of hospital physicians during their daily work. One way to handle social stressors with patients and their families can be found in reflecting these situations. Corresponding to Wilson [27], this could be realised by journaling, critical incident analysis, mentoring, and/or supervision. To reduce the frustration about how work needs to be done, it may be advantageous to introduce personalised training of key qualifications for handling administrative work. At the organisational level, it would be useful to improve staff planning and to reduce documentation and administrative work. One alternative could be to examine ways to facilitate the documentation and administrative tasks (e.g., easily manageable technical tools or more autonomy) and then to implement these. By reducing frustration about how work needs to be done, one could also reduce time pressure, as these two characteristics are closely correlated (*r* = .51, *p* < .001). The positive relationship between emotional dissonance (the suppression or control of the emotions) and emotional exhaustion has already been demonstrated for other professions, and high autonomy was shown to compensate the effects of high emotional dissonance [28]. For emotional exhaustion, problems in workflow due to other professional groups seem to be a relevant predictor. In order to reduce these problems, it seems to be useful to clarify the causes. There might be organisational reasons that agreements cannot be kept. But difficulties might also be caused by social conflicts, which have been found to be a frequent problem for assistant physicians [1]. In order to identify causes, interdisciplinary and cross-hierarchical working groups could be an adequate method [29] (e.g., focused on workflows and communication issues).

## Conclusions

The results of the current study demonstrated that hospital physicians experience certain work stressors differently with respect to their medical speciality. Therefore, stress prevention programs should consider these differences. Additionally, the meaning of time pressure, frustration about how work needs to be done, and emotional dissonance for aspects of health was illustrated. Aside longitudinal studies are needed in order to verify these cross-sectional findings; these results suggest some approaches for health promoting improvements.
